# Pancreatic Cancer: Molecular, Biochemical, Chemopreventive, and Therapeutic Aspects

**DOI:** 10.1100/tsw.2010.184

**Published:** 2010-10-01

**Authors:** Juan Iovanna, José Luis Neira

**Affiliations:** ^1^ INSERM U.624, Stress Cellulaire Parc, Scientifique et Technologique de Luminy, Marseille, France; ^2^ Instituto de Biología Molecular y Celular, Universidad Miguel Hernández, Elche (Alicante), Spain; ^3^ Instituto de Biocomputación y Física de Sistemas Complejos, Zaragoza, Spain

**Keywords:** metastasis, proteins, antibodies, drug-resistance, ubiquitination, pancreatic cancer, epithelial

## Abstract

Pancreatic cancer (PC) is the fourth leading cause of cancer death, with a median survival of 6 months and a dismal 5-year survival rate of 3—5%, a figure which has remained relatively unchanged over the past 25 years. PC is one of the most difficult diseases to treat due to late initial diagnosis and to resistance to the usual treatments. The presence of occult or clinical metastases at the time of diagnosis, together with the lack of effective chemotherapies, contributes to the high mortality in patients with PC. Its lethal nature stems from its propensity to disseminate rapidly to the lymphatic system and distant organs. Yet, understanding and stopping metastasis may prove to be one of the great potential strategies of treating PC. There is a dire need for the design of new and targeted therapeutic strategies that can overcome the drug resistance and improve the clinical outcome for patients diagnosed with the illness. The knowledge of the molecular aspects of PC is very important, and it is likely to be helpful in the design of newer drugs and the molecular selection of existing agents for targeted therapy. The inhibition of signal pathways can be carried out not only by small molecules, able to bind to selected regions of the target protein, but also by using large molecules as antibodies. The pathway to successful new therapies has been inhibited because of the rapidity with which agents tend to move into randomized, controlled trials without the extensive early testing necessary to optimize treatment regimens. However, lessons have been learned and our collective research effort has generated a substantial platform of knowledge from which further work will spring. The bioavailability of compounds such as antisense oligonucleotides and siRNAs in humans remains a big hurdle, which will require further improvement of gene-delivery strategies. Finally, the long-term goal of the therapy individualization for patients is possible if factors that predict treatment response, such as biological markers, could be determined accurately. These approaches are likely to comprise a mixture of targeted agents in combination with conventional chemotherapy and radiotherapy. For a clinically significant effect to be achieved, treatment strategies should either be in the form of (1) a “horizontal” approach, in which several oncogenic pathways (as those described in this series of reviews) are inhibited; or (2) a “vertical” approach, whereby multiple levels of a major pathway are targeted. Combination therapies, together with improved diagnostic tools and predictive markers, are ultimately desired in order to improve the bleak outlook for patients diagnosed with PC.

Juan Iovanna[1] and José Luis Neira[2,3]

Pancreatic cancer (PC) is the fourth leading cause of cancer death, with a median survival of 6 months and a dismal 5-year survival rate of 3–5%, and this figure has remained relatively unchanged over the past 25 years. Even for those patients diagnosed with local disease, the 5-year survival rate is only 15%. Thus, PC is one of the most difficult diseases to treat due to late initial diagnosis and to resistance to the usual treatments. The presence of occult or clinical metastases at the time of diagnosis, together with the lack of effective chemotherapies, contributes to the high mortality in patients with PC. Its lethal nature stems from its propensity to disseminate rapidly to the lymphatic system and distant organs. Moreover, PC is one of the most intrinsically drug-resistant tumors and the cancer cell resistance to chemotherapeutic agents is a major cause of its treatment failure. Gemcitabine (a nucleoside-based compound) is the standard chemotherapeutic drug for patients with an advanced illness state after a phase III trial in 1997. This trial demonstrated a modest survival advantage of this agent over 5-fluorouracil (median survival 5.65 vs. 4.41 months, respectively), but surprisingly this treatment improved alleviation of disease-related effects.

Known risk factors for the disease include cigarette smoking, chronic and hereditary pancreatitis, late-onset diabetes mellitus, and familial cancer syndromes. However, it has been estimated that more than two-thirds of human cancers, and among them PC, could be prevented by modification of lifestyle, including dietary modification. The dietary factors that are associated with increased risk of PC are meat, red meat in particular, and large intakes of high-energy food. Protection is mainly provided by fruit, vegetables, and vitamins. In recent years, more dietary compounds have been recognized as cancer chemopreventive agents because of their anticarcinogenic activity; therefore, early invasion and metastasis of PC could be preventable by these dietary compounds. For instance, the ingest of curcumin, found in a plant widely cultivated in tropical regions of Asia and Central America, and known by its pronounced anti-inflammatory, antioxidative, immunomodulating, antiatherogenic, and anticarcinogenic activities, modulates, among other proteins, the activity of NF-κB through inhibition of IKK activity in PC cells, and it further alters the expression of miRNAs in PC cells. Moreover, the dietary addition of genistein, a soy isoflavone found in soybeans and in most soy-protein products, inhibited NF-κB DNA-

binding activity, the Akt activity, and significantly down-regulated Notch signaling, leading to the inhibition of NF-κB and induction of apoptosis in PC cells. The intake of indole-3-carbinol, green tea catechins, lycopenes, and resveratrol (all of them present in several plants) has also been shown to be valuable in the prevention of PC. It is interesting to note that the experimental results with soy isoflavones, indole-3-carbinol, curcumin, and resveratrol appear to target similar signaling pathways, all of which are known to be involved in the development and progression of PC and, therefore, they are important targets for its prevention and/or treatment. However, it is important to keep in mind that primary prevention of PC is not feasible due to lack of identifiable risk factors. Notwithstanding this fact, existing knowledge provides sufficient information as to the novel application of several dietary or nutritional agents for the prevention of illness progression. In addition, these agents could be also useful for the treatment of PC, either as single agents or in combination, especially with existing therapeutic drugs.

Due to the absence of powerful treatments for PC, there is a dire need for the design of new and targeted therapeutic strategies that can overcome the drug resistance and improve the clinical outcome for patients diagnosed with the illness. To this end, the knowledge of the molecular aspects of PC is very important, and it is likely to be helpful in (1) the design of newer drugs and (2) the molecular selection of existing agents for targeted therapy. In the papers comprising this series devoted to PC, several leading groups from different world-wide laboratories have summarized what we know regarding the molecular aspects of the illness, and how some of those molecular pathways could be exploited for the prevention and/or treatment of PC. The several reviews tackle different aspects of PC, from the new antibody (AB) therapies to the more ground-layer aspects of structural biology, or encompassing the importance of metastasis in PC, to the influence of ubiquitylation in the development of the illness.

Intensive investigation of molecular pathogenesis will aid in identifying useful molecules for diagnosis, treatment, and prognosis of PC. In the past several years, considerable research has focused on identifying molecular events in PC, and their correlation with the clinic-pathological status. It has been found that multiple subsets of genes undergo genetic changes, either activation or inactivation, during the development and progression of PC. The activation of oncogenes and the inactivation of tumor-suppressor genes are partly responsible for the initiation and progression of PC. Moreover, the deregulation of molecules in several cell signaling pathways, such as EGFR (epidermal grow factor receptors), Akt, NF-κB, CCKR (chemokine receptors), Wnt, FADK (focal adhesion kinase), Notch, or Hedgehog, and their molecular cross-talks, also play important roles in the molecular pathogenesis of PC. The structures of the most important proteins intervening in these pathway signaling routes are described in the paper by Bravo and Neira[1]. Mutations, which appear more frequently in PC, lead to structural changes in the proteins involved, hampering the cross-talk with other proteins and, then, resulting into inactivation. This inactivation is especially important in several tumor-suppressor genes (as in p16, SMAD4, or p53 pathways). Furthermore, the knowledge of the atomic details of the structure provides us with new arms to design more efficient drugs. For instance, in EGFR signaling, the phosphorylation of EGFR activates molecules in different cell signaling pathways, including the PI3K, Src, MAPK, and STAT; these pathways are involved in cell cycle progression, cell division, survival, motility, invasion, and metastasis. Knowing how and where (i.e., which residues are involved) phosphorylation at the molecular level occurs has led to the design of erlotinib (Tarceva®), a small molecule inhibitor of the EGFR tyrosine kinase. Experimental studies have shown that erlotinib inhibits EGFR tyrosine kinase activity and cell growth in PC cell lines and in an animal model. In a phase III trial for patients with advanced PC, erlotinib together with gemcitabine has shown a statistically significant survival benefit compared with gemcitabine alone. In another study combining erlotinib with gemcitabine and irradiation for locally advanced unresectable PC, most of the examined patients showed disease stabilization. Finally, when combined with capecitabine, erlotinib has shown considerable antitumor activity and better tumor control in a phase II trial for advanced PC.

The inhibition of signal pathways can be carried out not only by small molecules, able to bind to selected regions of the target protein, but also by using large molecules as ABs. This is the topic described by Chames and coworkers[2]. The VEGF (vascular endothelial growth factor) protein, which is involved in angiogenesis during tumor growth and dissemination, is one of the first examples where an AB (called bevacizumab) has been designed to hamper binding to its receptor (VEGFR). EGFR is the other example described by Chames and colleagues. Cetuximab, a monoclonal AB that targets EGFR, binds to EGFR competitively with high affinity, preventing activation of EGFR by its ligands. By binding to EGFR, cetuximab inhibits cell proliferation, enhances apoptosis, and reduces angiogenesis and invasion. Cetuximab is currently under investigation in PC; the combination of cetuximab and gemcitabine showed promising activity against an advanced illness and improved survival in animal study, but it was ineffective in a phase III trial in patients with locally advanced and metastatic PC. No objective responses were seen in phase II trials of cetuximab in combination with gemcitabine and intensity-modulated radiotherapy. Trastuzumab, an anti-HER-2/neu AB, showed cell growth inhibitory activity against human PC cell lines and antitumor activity in an orthotopic mouse model. Mesothelin and carcinoembryonic antigens are two other proteins where initial steps towards the design of ABs have been given. Preclinical studies and the design of new ABs against the epithelial cell adhesion, nonspecific cross-reacting antigen, mucin-1, or death receptor 5 have also shown promising results; they induce, among other effects, apoptosis via caspase activation.

It has been indicated above that by hampering phosphorylation, we are able to alter several signaling pathways where EFGR intervenes, but this is not the sole protein modification that can have dramatic results in PC development. Soubeyran and colleagues describe the importance of ubiquitylation[3]. Among proteins affected by a defective ubiquitylation are SMAD4 (where mutation of a conserved arginine increases its polyubiquitylation), transglutaminase, or Pim-1 (a proto-oncogene regulating apoptosis). Furthermore, Souberyan and coworkers show us how in PC, some types of proteasome-machinery-related proteins are accumulated. Thus, the proteasome could be considered a promising target for PC treatment; the authors describe the new proteasome inhibitors with interesting pharmacological applications, such as bortezomib (Velcade®, PS-341), which could lead to activation of apoptotic programs by binding to the proteasome in a reversible manner.

Metastasis is one of the great potentials of PC (together with its strong drug resistance). Understanding metastasis requires the knowledge of cellular resistance to the stress occurring during cellular escape and the colonization of the tissue. The paper by Cano and Iovanna[4] describes the model for stress resistance in PC, the 88-residue long protein, p8. This protein is involved in a plethora of functions, such as the resistance towards a PC cell response, the remodeling on chromatin, and the regulation of cell cycle. The mainly disordered structural character of p8 and its acquisition of secondary structure upon binding to other proteins make p8 a potential target for the design of new drugs. On the other hand, the basis of the highly metastatic potential of PC cells is a physiological process called the epithelial-to-mesenchymal transition (EMT). This process allows the conversion of adherent epithelial cells (which are tightly bound in an organized healthy tissue) into the independent fibrobrastic cells, able to invade the extracellullar matrix and to migrate to other tissues. The features of this transition and the intervening proteins are described by Iovanna and colleagues[5]. The main EMT-triggering factors in the PC are the hepatocyte growth factor (which also intervenes in several signaling cascades), the transforming growth factor, and the bone morphogenetic protein. There are also several signaling pathways, such as SMAD4 and Ras routes, as well as chromatin-remodeling elements (the histone deacetylase and the High Mobility Group A proteins), that are also involved in EMT. Furthermore, short noncoding RNAs implicated in post-translational regulation of gene expression, called microRNAs, also intervene in EMT. EMT-mediated modulation of miRNAs seems to be associated with down-regulation of five members of the miRNA family, and that regulation appears at the basis of the protumoral attributes of EMT.

All these studies have shown us that the development and progression of PC are closely associated with the activation of oncogenes; the inactivation of tumor-suppressor genes; and the deregulation of EGFR, Akt, NF-κB, and their downstream signaling pathways. Although targeted therapies for PC have yielded encouraging results *in vitro* and in animal models, these findings have not been translated to improved outcomes in clinical trials. Reasons for this failure might include an incomplete understanding of the biology of PC, the selection of poor active agents, problems with the trial design (such as inappropriate therapeutic end points or patient selection), and the rapidity with which agents move into randomized, controlled trials without the extensive early testing necessary to optimize treatment regimens. Furthermore, preclinical studies performed on mouse models do not always recapitulate the human condition, which is a particular problem with human PC xenografts in immunodeficient mice. Despite these setbacks, lessons have been learned and our collective research effort has generated a substantial platform of knowledge from which further work could spring. Genetically engineered immunocompetent mice, such as those with KRAS or TP53 mutations, have been developed and they hold promise for the future studies of the disease. The bioavailability of compounds such as antisense oligonucleotides and siRNAs in humans remains a big hurdle, which will require further improvement of gene-delivery strategies. Finally, the long-term goal of the therapy individualization for patients is possible if factors that predict treatment response, such as biological markers, could be determined accurately. These approaches are likely to comprise a mixture of targeted agents in combination with conventional chemotherapy and radiotherapy. For a clinically significant effect to be achieved, treatment strategies should either be in the form of (1) a “horizontal” approach, in which several oncogenic pathways (as those described in this series of reviews) are inhibited; or (2) a “vertical” approach, whereby multiple levels of a major pathway are targeted. Combination therapies, together with improved diagnostic tools and predictive markers, are ultimately desired in order to improve the bleak outlook for patients diagnosed with PC.
